# Genome-Wide Identification and Evolution-Profiling Analysis of TPS Gene Family in *Triticum* Plants

**DOI:** 10.3390/ijms25158546

**Published:** 2024-08-05

**Authors:** Yiyang Liu, Dongyang Li, Yue Liu, Jiazheng Wang, Chang Liu

**Affiliations:** 1 College of Agronomy, Jiangxi Agricultural University, Nanchang 330045, China; yiyangliu0220@163.com; 2 College of Agronomy, Shenyang Agriculture University, Shenyang 110866, China; lidongyang0713@163.com; 3 Institute of Applied Ecology, Chinese Academy of Sciences, Shenyang 110866, China; 4 College of Agronomy, Sichuan Agricultural University, Chengdu 611130, China; wangjiazheng0918@163.com

**Keywords:** *Triticum* plants, TPS gene family, evolution, gene duplicated events, RNA-seq

## Abstract

Terpenoids play a crucial role in plant growth and development, as well as in regulating resistance mechanisms. Terpene synthase (TPS) serves as the final step in the synthesis process of terpenoids. However, a comprehensive bioinformatics analysis of the TPS gene family in *Triticum* plants had not previously been systematically undertaken. In this study, a total of 531 TPS members were identified in *Triticum* plants. The evolutionary tree divided the TPS proteins into five subfamilies: Group1, Group2, Group3, Group4, and Group5. The results of the duplication events analysis showed that TD and WGD were major driving forces during the evolution of the TPS family. The cis-element analysis showed that the TPS genes were related to plant growth and development and environmental stress. Moreover, the GO annotation displayed that the biological function of TPS was relatively conserved in wheat plants. The RNA-seq data showed that the rice and wheat TPS genes responded to low-temperature stress and exhibited significantly different expression patterns. This research shed light on the functions of TPSs in responding to abiotic stress and demonstrated their modulatory potential during root development. These findings provide a foundation for further and deeper investigation of the TPSs’ functions in *Triticum* plants.

## 1. Introduction

Terpenoids, also known as isoprenoids, represent a diverse class of natural products characterized by their intricate structures. To date, more than 80,000 terpenoids and their derivatives have been identified in various organisms, such as bacteria, fungi, insects, and plants [1,2]. Terpenoids are prevalent across organisms, with green plants, especially flowering varieties, displaying a remarkable terpenoid diversity that is crucial to both primary and secondary metabolism [3,4]. While a minority of terpenoids contribute to primary metabolism, including hormones, electron transport components, protein modifiers, membrane regulators, and antioxidants, they all play vital roles in sustaining life processes. For example, carotenoids aid photosynthesis [4]; hormones like gibberellins, abscisic acid, and cytokinin regulate growth; phytosterols affect membrane integrity; ubiquinone and plastoquinone facilitate electron transport; and polyphenols participate in protein glycosylation [5,6,7].

Terpene synthase (TPS) serves as the key enzyme responsible for sesquiterpene synthesis [8,9]. In higher plants, TPS genes often exist in families and have garnered increasing attention with the advancement of modern sequencing technologies. The TPS gene family was identified in *Arabidopsis thaliana*, *Oryza sativa*, *Brachypodium distanchyon*, maize, and potato [10,11,12,13,14]. Meanwhile, a comprehensive study that utilized genomic and transcriptome data in Dendrobium officinale identified a total of 34 TPS genes, which were further classified into four subfamilies. These genes exhibited predominant expression in flowers, followed by roots and stems [9]. In the genome of Camellia, 80 TPS genes were discovered, where most *CsTPS* genes showed enhanced transcriptional activity upon treatment with methyl jasmonate [15]. Therefore, TPS proteins are necessary for plant growth and development, as well as enhancing resistance to abiotic and biotic stress. The whole-genome identification of TPS protein is practicable for us to enable plant tolerance. However, the understanding of TPS proteins in wheat is lacking.

In this study, we conducted an investigation of the TPS gene family in wheat plants by employing Arabidopsis, which is a dicotyledonous model plant, and rice, which is a monocotyledonous model plant. We employed various analyses, including phylogenetic tree construction, promoter analysis, functional annotation using Gene Ontology (GO), identification of duplication events, protein interaction network analysis, and an examination of the expression patterns under low-temperature stress for the TPS genes. Moreover, we explored the evolutionary process of the TPS gene family in wheat species. Our study successfully identified the repertoire of TPS genes in wheat plants, thus shedding light on the evolution and genome duplication events specific to wheat. Furthermore, our findings establish a foundation for further investigations into the evolutionary origin and functional roles of TPS genes in wheat plants. Additionally, the results of our study provide valuable insights into studying the genes associated with stress responses in wheat plants, thus serving as a useful reference for future research.

## 2. Results

### 2.1. Identification of TPS Family Members in Triticum Plants

**Figure 1:**
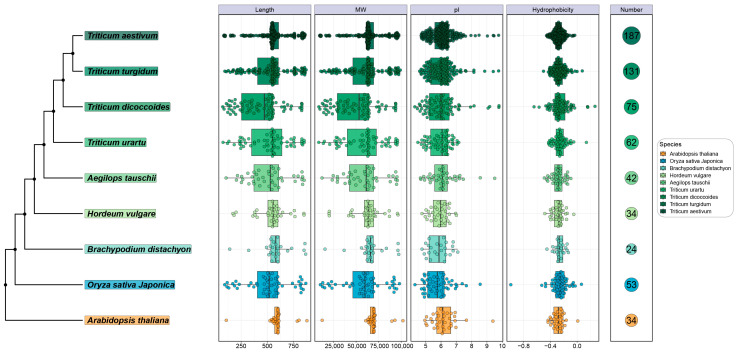
Characteristics of TPS family members in 9 species, including protein length, molecular weight, isoelectric point, and hydrophilicity.

In the nine plant genomes involved in this study, 24–187 TPS genes were confirmed by HMMER 3.2.1 and BLAST 2.9.0+ software, of which 34 were identified in Arabidopsis thaliana, 53 in rice, 24 in Brachypodium distichum, 34 in Hordeum vulgare, and 42–187 were identified in the remaining five *Triticum* plants (42 in *Aegilops tauschii*, 75 in *Triticum dicoccoides*, 131 in *Triticum turgidum*, 187 in *Triticum aestivum*, and 62 in *Triticum urartu*). Additionally, our investigation revealed that the number of subfamily members within the TPS family in *Triticum* plants exhibited an increase corresponding to the augmentation of *Triticum* plant genomes. This finding indicates that the expansion of the TPS family might be associated with genome-doubling events in *Triticum* plants. The protein sequence length of all the TPSs was less than 878 amino acids and ranged from 74 to 878; the molecular weight ranged from 8.6 to 101.9 kDa; and the theoretical PI ranged from 4.28 to 9.79. And the predicted subcellular localization of all TPS proteins was in the chloroplast (Figure 1, Supplemental Data S1). Overall, the number of TPS members gradually increased with the increase in the genome size. These results revealed basic information about the TPS genes and suggest that they may have different functional potentials in chloroplasts.

### 2.2. Phylogenetic Analysis of TPSs

**Figure 2:**
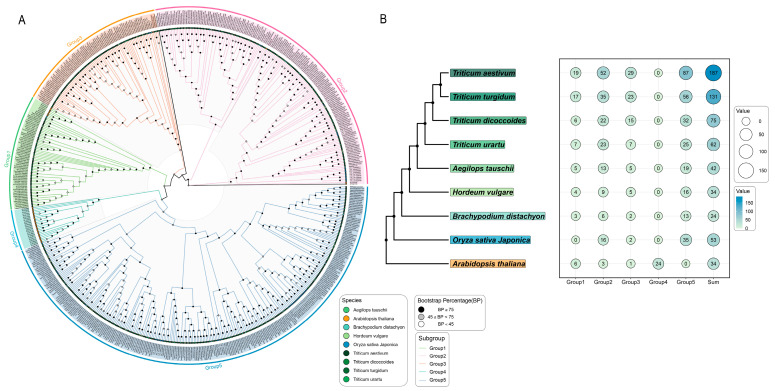
Phylogenetic tree of TPS genes in 9 species. (**A**) The evolutionary tree was constructed by the maximum likelihood method (bootstrap values: 1000 replicates) using IQ-TREE. The tree was visualized using the R package ggtree. Green, orange, pink, sky blue, and blue represent the subgroups of Group1, Group2, Group3, Group4, and Group5, respectively. (**B**) Statistical results of different subfamilies in different species.

To further elucidate the evolutionary relationship of *TaTPS*s, a phylogenetic tree containing 642 TPS genes in nine species was constructed (Figure 2). TPS genes could be grouped into Group1, Group2, Group3, Group4, and Group5 based on the Arabidopsis and bootstrap values in the phylogenetic tree. Among them, Group1, Group2, and Group3 were commonly found in dicotyledonous and monocotyledonous species. However, Group4 was exclusively identified in Arabidopsis, while Group5 was only observed in monocotyledonous plants. These findings suggest that these two subfamilies likely originated during the evolutionary divergence of monocotyledonous and dicotyledonous plants.

### 2.3. Duplication Events Analysis of TPS Genes in Triticum Plants

**Figure 3:**
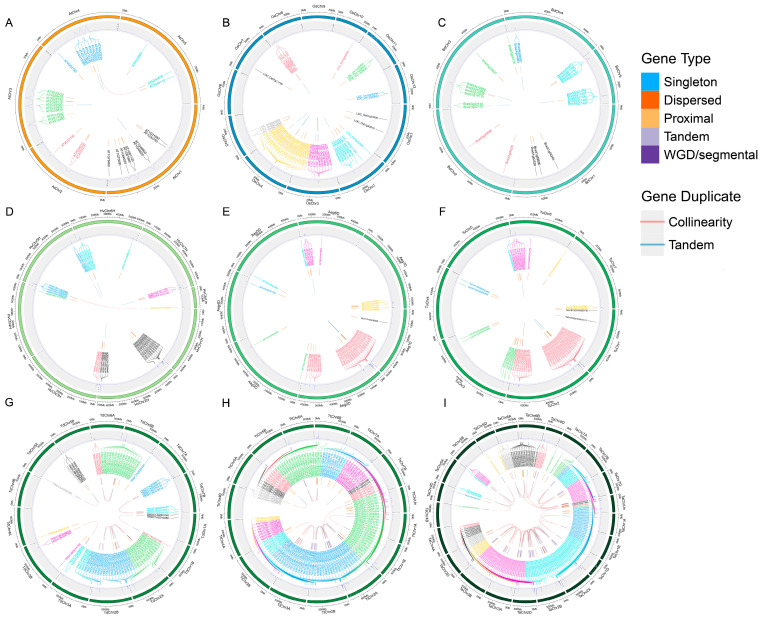
The chromosome location and duplicated gene pair of TPS genes in 9 species: (**A**) *Arabidopsis thaliana*; (**B**) *Oryza sativa Japonica*; (**C**) *Brachypodium distachyon*; (**D**) *Hordeum vulgare*; (**E**) *Aegilops tauschii*; (**F**) *Triticum urartu*; (**G**) *Triticum dicoccoides*; (**H**) *Triticum trugidum*; (**I**) *Triticum aestivum*. The duplicate gene types are displayed in different colors. Whole-genome duplication (WGD) and tandem duplication (TD) events are shown in red and blue, respectively.

In this study, the physical locations of the TPS genes were extracted based on the reference genome annotation files of nine poaceae plants. The results show that the distribution of the TPS genes on the chromosomes was uneven in *Arabidopsis thaliana*, *Oryza sativa*, and *Brachypodium distachyon*. This suggests that there was no specific preference for the chromosomal distribution of the TPS genes in these species. In wheat plants, the TPS genes were also randomly distributed on the chromosomes; however, the TPS genes were found to be more concentrated at both ends of the chromosomes, which appeared in the form of gene clusters (Figure 3). This observation may be attributed to the evolutionary history of wheat plants and their ancestral species.

The tandem duplication (TD) and whole-genome duplication (WGD) of genes drive the evolution and expansion of the gene family [16,17,18]. We analyzed the duplication events of TPS genes in nine species. In *Arabidopsis thaliana*, *Oryza sativa*, and *Brachypodium distachyon*, 5, 4, and 10 pairs of TD genes and 1, 0, and 0 pairs of WGD were found, respectively. These duplicated genes accounted for 32.3%, 33.3%, and 32.1% of the total TPS genes, respectively (Figure 3A–C). The above results indicate that TD led to the expansion of the TPS gene. In *Triticum* plants, 4, 6, 10, 17, 27, and 40 pairs of TD genes and 1, 2, 0, 14, 25, and 87 WGD gene pairs were found in *Hordeum vulgare*, *Aegilops tauschii*, *Triticum urartu*, *Triticum dicoccoides*, *Triticum trugidum*, and *Triticum aestivum*, respectively. These duplicated genes accounted for 29.4%, 31%, 27.4%, 64%, 64.1%, and 74.9% of all the TPS genes, respectively (Figure 3D–I). These results suggest that during wheat evolution, TD and WGD largely contributed to the expansion of the TPS genes.

Meanwhile, we counted the Ka, Ks, and Ka/Ks values of the duplication gene pairs using KaKs_Calculator to analyze the evolutionary selection of duplication pairs in the TPS gene family. We found that the Ka/Ks ratios of most gene pairs were less than 1 (Supplemental Data S2, Supplemental Figure S1), implying these TPS*s* underwent negative selection. Only two gene pairs, *TraesCS2A02G137900*–*TraesCS2D02G140900* and *TraesCS2D02G008600*–*TraesCS2D02G008700* (Supplemental Data S2), had Ka/Ks ratios greater than 1, indicating that two duplicated gene pairs may undergo positive selection and they are important for the evolution of *Triticum aestivum*.

### 2.4. Collinearity Analysis of TPSs in Triticum Plants

**Figure 4:**
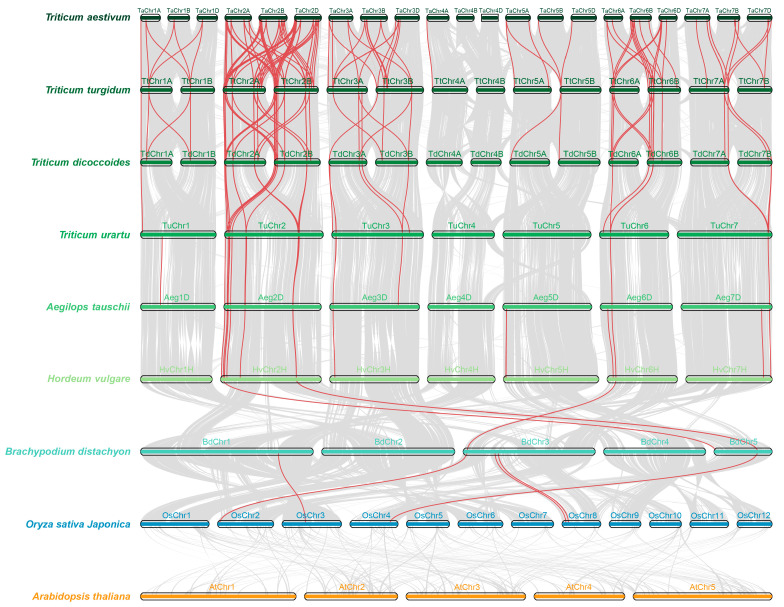
Syntenic analysis of the TPS genes between 9 species. The collinear blocks are shown by gray lines, while the syntenic TPS homologous gene pairs are highlighted by red lines. The species from top to bottom are *Triticum aestivum*, *Triticum trugidum*, *Triticum dicoccoides*, *Triticum urartu, Aegilops tauschii*, *Hordeum vulgare*, *Brachypodium distachyon*, *Oryza sativa Japonica*, and *Arabidopsis thaliana*.

To more deeply investigate the evolution mechanisms of TPS genes, we analyzed the syntenic relationships between nine species. As shown in Figure 4, there were no collinear TPS gene pairs in Oryza sativa and Arabidopsis thaliana. Moreover, 5, 3, 16, 18, 38, 73, and 145 ortholog TPS gene pairs were identified when we compared *Oryza sativa* with *Brachypodium distachyon*, *Hordeum vulgare*, *Aegilops tauschii*, *Triticum urartu*, *Triticum dicoccoides*, *Triticum turgidum*, and *Triticum aestivum*, respectively. Among the remaining poaceae plants, there were 5, 3, 16, 18, 38, 73, and 145 collinear TPS gene pairs, respectively (Figure 4). The above results indicate that the TPS genes existed before the evolution of dicotyledonous and monocotyledonous plants. At the same time, the number of collinear TPS gene pairs continued to increase with the evolution of wheat plants. In addition, we also found that chromosomes 2, 3, 6, and 7 of *Hordeum vulgare*, *Aegilops truncatula*, and *Triticum urartu* contained abundant TPS colinear gene pairs. Moreover, *Triticum* dicoccoides (1AB, 2AB, and 3AB), *Triticum trugidum* (6AB and 7AB), and *Triticum aestivum* (1ABD, 2ABD, 3ABD, 6ABD, and 7ABD) were abundant in TPS colinear gene pairs. This result may provide a reliable basis for chromosome duplication and assembly during *Triticum* evolution.

We also analyzed the Ka, Ks, and Ka/Ks values of the abovementioned collinear gene pairs. We found that the Ka/Ks ratios of most collinear gene pairs were less than 1 (Supplemental Data S3 and Supplemental Figure S2), implying these TPSs underwent negative selection in the process of *Triticum* evolution. However, during the evolution of *Triticum aestivum* and *Triticum turgidum*, *Triticum turgidum* and *Triticum dicoccoides*, *Triticum dicoccoides* and *Triticum urartu*, *Triticum urartu* and *Aegilops tauschii*, 3, 4, 1, and 1 colinear gene pairs had Ka/Ks values greater than 1, respectively (Supplemental Data S3), indicating that these collinear gene pairs may undergo positive selection. The abovementioned TPS genes were retained during the evolution of *Triticum* plants and may have contributed to their growth and development, as well as resistance to adverse stress, during the polyploid evolution process.

### 2.5. Cis-Elements Analysis of TPS Family in Triticum Plants

**Figure 5:**
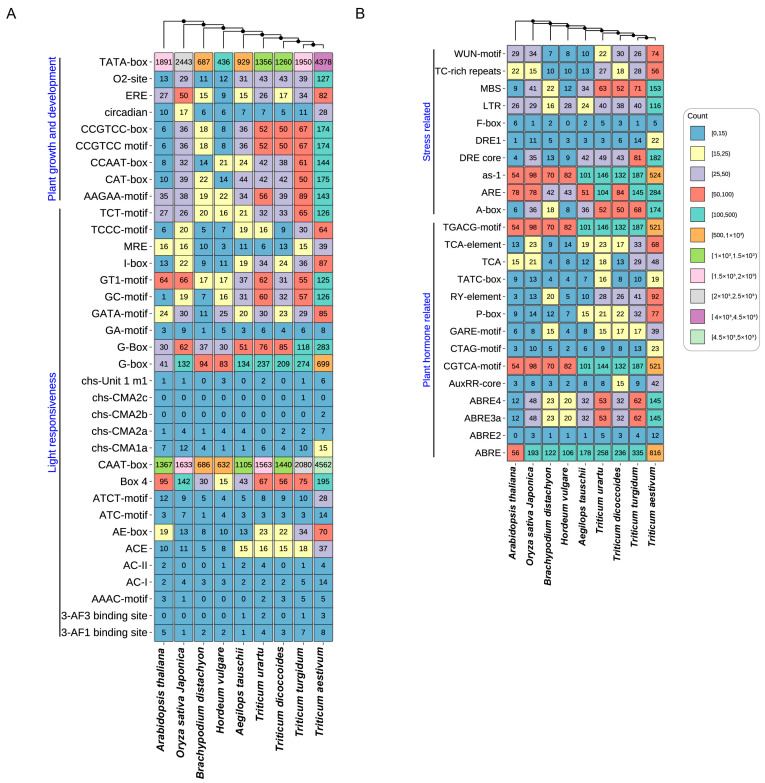
Cis-elements analysis of TPS genes in 9 species. (**A**) Cis-elements related to plant growth and development and light responsiveness. (**B**) Stress-related and plant-hormone-related cis-elements.

To predict the functions and regulatory mechanisms of TPS genes in the nine species, the cis-elements within their promoter were analyzed. A total of 59 different cis-elements in the promoter of the TPS genes were detected (Figure 5, Supplemental Data S4). And these included cis-elements involved in plant growth and development (TATA-box, CAT-box, CCAAT-box, etc.), light responsiveness (G-Box, Box 4, ACE, etc.), stress-related (WUN-motif, LTR, DRE core, ARE, etc.), and plant-hormone-related factors (ABREm TCA-element, AuxRR-core, GARE-motif, etc.). These findings suggest that TPS genes likely interact with transcription factors involved in diverse physiological processes, encompassing plant development, light perception, stress adaptation, and hormonal regulation. It is plausible to infer that the TPS genes may play a significant role in regulating the aforementioned physiological processes. Interestingly, *Oryza sativa* and *Arabidopsis thaliana* contained more WUN-motif elements than *Aegilops tauschii*, *Hordeum vulgare*, and *Brachypodium distachyon*. Moreover, *Triticum aestivum*, *Triticum trugidum*, *Triticum dicoccoides,* and *Triticum urartu* contained more MBS elements than *Aegilops tauschii*, *Hordeum vulgare*, *Brachypodium distachyon*, *Oryza sativa Japonica*, and *Arabidopsis thaliana*. The results suggest that different species may have different potential roles for TPS genes. Furthermore, our research findings indicate that the number of promoters in wheat plants demonstrated a substantial increase during the evolutionary process, which coincide with genome replication events. This observation strongly suggests that the occurrence of genome doubling likely contributed to the expansion of the promoter regions. Consequently, it can be hypothesized that the TPS genes in wheat plants might possess an increased repertoire of biological functions, enabling them to effectively respond to diverse environmental changes.

### 2.6. Interaction Network of TPS Proteins

**Figure 6:**
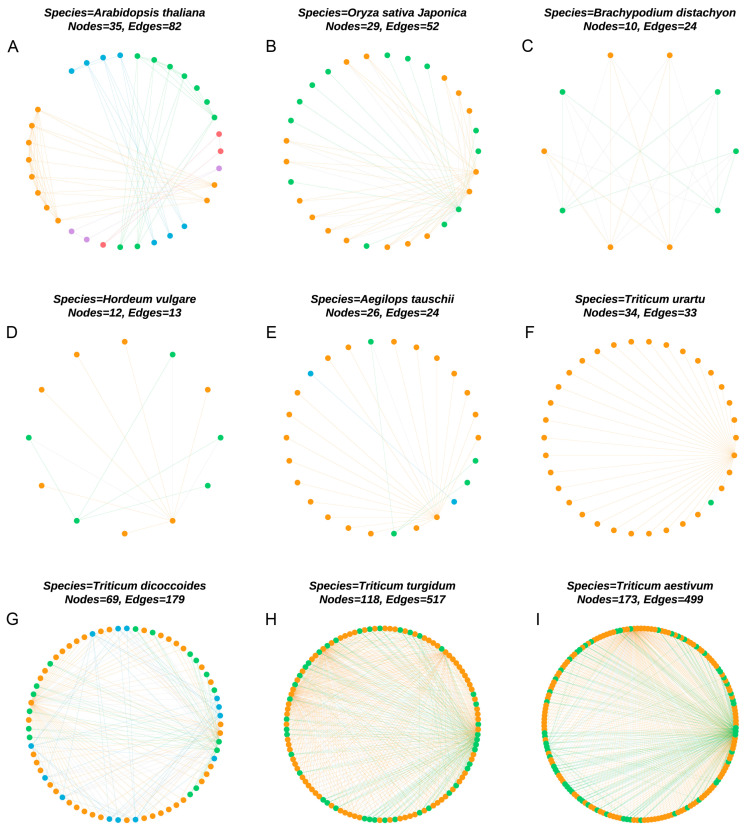
The protein–protein interaction network of TPS proteins in 9 species: (**A**) *Arabidopsis thaliana*; (**B**) *Oryza sativa Japonica*; (**C**) *Brachypodium distachyon*; (**D**) *Hordeum vulgare* (**E**) *Aegilops tauschii*; (**F**) *Triticum urartu*; (**G**) *Triticum dicoccoides*; (**H**) *Triticum trugidum*; (**I**) *Triticum aestivum*. Different colors represent different network modules.

In order to further clarify the functions and regulatory pathways among the TPS family, a protein–protein interaction network was analyzed and predicted by the AraNet2 database [19] (Figure 6). The results of our study reveal distinct protein interaction networks among the TPS proteins from the nine species included in our analysis. The TPS interaction network of Arabidopsis thaliana was found to comprise five distinct modules. In contrast, monocots were observed to have only two to three modules within their TPS interaction networks. This discrepancy suggests that there may be variations in the complexity and organization of TPS protein interactions between dicotyledonous (such as *Arabidopsis thaliana*) and monocotyledonous plants. Furthermore, it was observed that during the genome-doubling process in *Triticum* plants, both the number of nodes and edges within the protein interaction network of the TPS family exhibited a continuous increase. This suggests that the complexity of the network became progressively greater as a result of the genome duplication events. These findings indicate that the protein interaction relationships within the TPS family became increasingly intricate during the evolutionary history of wheat. This enhanced complexity may potentially contribute to the growth, development, and improved stress resistance of polyploid *Triticum* plants.

### 2.7. Gene Ontology (GO) Functional Annotation of TPSs in Triticum Plants

**Figure 7:**
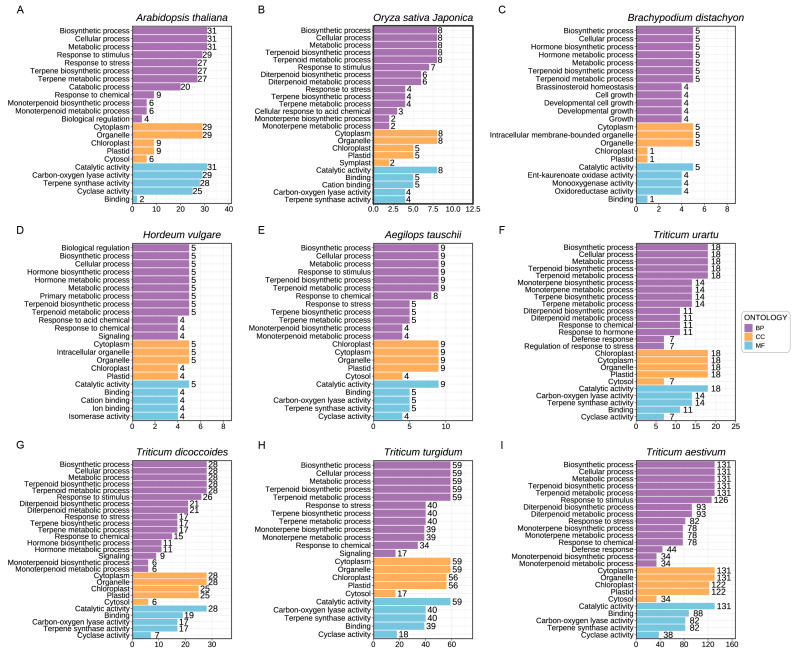
GO functional annotations of TPSs in *Triticum* species (**A**) *Arabidopsis thaliana*; (**B**) *Oryza sativa Japonica*; (**C**) *Brachypodium distachyon*; (**D**) *Hordeum vulgare* (**E**) *Aegilops tauschii*; (**F**) *Triticum urartu*; (**G**) *Triticum dicoccoides*; (**H**) *Triticum trugidum*; (**I**) *Triticum aestivum*. Gene Ontology terms were divided into three categories: biological process (BP), cellular components (CCs), and molecular function (MF). The vertical axis shows the annotated GO terms. The horizontal axis shows the number of genes corresponding to the GO Terms. Purple, orange, and blue indicate BPs, CCs, and MFs, respectively.

In order to further explore the potential biological functions of TPS, the eggNOG-mapper database [20] was used to perform GO functional annotation on the TPS protein sequences of nine Poaceae plants. Subsequently, a comprehensive statistical analysis was performed on these annotations (Figure 7). The key findings are summarized as follows: among the biological process (BP) categories, the TPS proteins in these nine plants were implicated in biosynthetic and cellular processes, as well as responses to stimuli. In terms of cellular components (CCs), they were associated with the cytoplasm, organelles, and chloroplasts. Lastly, within molecular function (MF), the proteins exhibited catalytic activity, specifically carbon–oxygen lyase activity and terpene synthase activity, among others.

The results obtained from our study suggest that the biological functions of TPS genes are highly conserved between monocots and dicots, indicating that they may perform similar roles in both plant groups. However, in *Triticum* plants, with the occurrence of genome duplication events, we observed an increase in the number of TPS genes, alongside a broader range of biological functions associated with these genes. This finding further reinforces the notion that the TPS gene plays a crucial role in the process of genome duplication and expansion in *Triticum* plants.

### 2.8. The Expression Analysis of TPSs in RNA-Seq

To investigate the potential biological functions of TPS genes in response to abiotic stress, specifically low temperature, we analyzed the expression patterns of TPS genes in rice and wheat using transcriptome data. By examining the transcriptome profiles, we aimed to uncover how the expression of TPS genes was modulated under low-temperature conditions and determined their potential roles in mediating stress responses in these two crops (Supplemental Data S5). In the transcriptome data of rice under low-temperature treatment, the experiment involved subjecting rice plants to low-temperature conditions for different durations: 1 day, 2 days, 3 days, and 4 days. Among these conditions, it was found that 20 *OsTPS* genes were expressed under the low-temperature treatment. Furthermore, it was observed that 60% of the *OsTPS* genes exhibited a continuous decrease in their expression levels as the duration of the low-temperature treatment increased. On the other hand, the expression levels of the remaining 40% of *OsTPS* genes gradually increased with continuous low-temperature treatment. These findings suggest that the expression of *OsTPS* genes was dynamically regulated in response to prolonged exposure to low-temperature conditions in rice.

In the transcriptome data of wheat under the low-temperature treatment, the experiment involved exposing wheat plants to a range of low temperatures, including −25, −20, −15, −10, −5, 0, and 5 °C. Among these conditions, it was observed that 91 *TaTPS* genes were expressed under the low-temperature treatment. Furthermore, when examining the expression levels of these *TaTPS* genes, it was found that 51 genes exhibited their highest expression levels at −25 and −20 °C, indicating their specific response to ultra-low temperatures. Conversely, 33 genes showed their highest expression levels at 0 and 5 °C, representing their response to relatively milder low temperatures. Most of the TPS genes did not exhibit significant changes in expression levels at −15, −10, and −5 °C. These results suggest that *TaTPS* genes may play a crucial role in responding to and coping with ultra-low-temperature and low-temperature conditions in wheat. Their differential expression patterns indicate their potential involvement in key biological functions related to cold stress adaptation in *Triticum* plants.

In summary, our findings indicate that TPS genes in both rice and wheat responded to low-temperature stress. However, the specific patterns of the response to low temperatures differed between these two species. This suggests that TPS genes may play distinct biological functions in coping with adverse stresses in different plant species. Further research is needed to elucidate the precise roles of TPS genes in stress responses and their potential contributions to the adaptation and survival of plants under adverse conditions.

## 3. Discussion

Terpenoids play a critical role in enhancing plant resistance, and terpene synthases are key contributors to the structural diversity of these natural compounds [21]. They catalyze the conversion of prenyl diphosphates into various forms, including volatile monoterpenes, sesquiterpenes, semi-volatile compounds, and non-volatile diterpenes [22]. Therefore, it is of utmost importance to study the gene family responsible for terpenoid synthesis. Although TPS genes were identified in many species, including *Arabidopsis thaliana*, *Oryza sativa*, *Brachypodium distanchyon*, maize, and potato [10,11,12,13,14], the TPS gene family in *Triticum* plants had not previously been comprehensively analyzed. In this study, we completely identified 531 TPS genes in *Triticum* plants and investigated their phylogenetic relationships, gene duplication events, cis-elements in the promoter sequence, and expression patterns under the cold response of the TPS gene family.

The TPS gene was first reported in Arabidopsis (Aubourg et al., 2002), and subsequent studies showed that TPS is widely found in plants, such as 53 in rice [11], 8 in potato [12], 34 in d. officinale [9], 80 in camellia [15], and 16 in *A. hypogaea* [23]. Here, 33, 53, 24, 34, 42, 75, 131, 187, and 62 TPS genes were identified in *Arabidopsis thaliana*, rice, *Brachypodium distichum*, *Hordeum vulgare*, *Aegilops tauschii*, *Triticum dicoccoides*, *Triticum turgidum, Triticum aestivum*, and *Triticum urartu*. Obviously, the members of TPS genes in different plants varied widely. It is worth noting that the number of TPS genes (62) in *Triticum urartu* was much less than that in *Triticum turgidum* (131) and *Triticum aestivum* (187) (Figure 1). We hypothesized that the smallest genome of *Triticum urartu* among the rice species might (at least partially) account for the difference.

The phylogenetic analysis showed that based on the bootstrap values of the evolutionary tree and Arabidopsis thalian, the TPS genes of the 531 genes could be divided into five groups (Figure 2). Group4 only existed in the dicotyledonous plants, and Group5 only existed in the monocotyledons. Our results contribute to the understanding of TPS gene evolution in monocotyledons and dicotyledons. In addition, with the evolution of *Triticum* plants, the number of TPS family members has continued to increase. This suggests that TPS may play a potential biological function in the process of polyploidization in *Triticum* plants. Polyploid plants contain more abundant biomass and a stronger ability to resist adverse stress than diploid plants [24]. Therefore, the TPS gene family may be more conducive to promoting plant growth and development or responding to adversity.

Gene duplication events in plants are the main factor causing gene family expansion [25]. These duplication events are not only the driving force for the expansion of plant gene families but also an important factor in improving the ability of plants to resist stress [26,27,28]. In *Triticum* plants, these duplicated TPS genes accounted for 29.4%, 31%, 27.4%, 64%, 64.1%, and 74.9% of all the TPS genes (Figure 3D–I). In summary, phylogenetic patterns and gene duplication events are the main driving forces for the difference in the number of TPS families in *Triticum* plants. In addition, during the evolution of *Triticum* plants, genome doubling and chromosome duplication occurred. The TPS gene family experienced a large amount of expansion during the evolution of *Triticum* plants rather than shrinking or being lost, indicating the importance of TPS in the evolutionary process.

In addition, we analyzed the promoter cis-acting elements of the TPS genes and found that the cis-elements of the promoter of the TPS gene may be involved in plant growth and development, stress-related, light responsiveness, and plant-hormone-related factors. And because the number of promoter cis-acting elements has been increasing with the evolution of *Triticum* plants, it shows that TPS may have more biological functions in polyploid *Triticum* plants.

**Figure 8:**
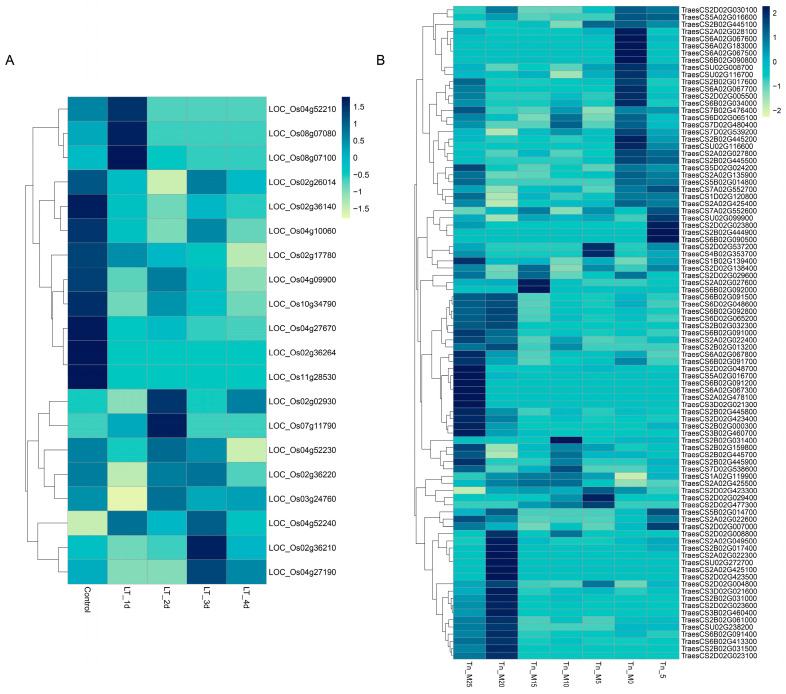
The expression profiles of *OsTPSs* (**A**) and *TaTPSs* (**B**) under cold treatment. The data are shown in a heatmap with gene expression in different treatments with row-scaled FPKM values.

A series of studies showed that the TPS gene plays an important role in plant cold tolerance, such as *AhTPS9* overexpression improving cold tolerance by protecting the photosynthetic system of plants and regulating sugar-related metabolites and genes [23]. The overexpression of *Triticum aestivum TaTPS11* in *Arabidopsis* enhanced the cold tolerance, suggesting its potential value in wheat cold-tolerance breeding [29]. We explored the expression patterns of rice and wheat TPS genes under low-temperature stress. The results showed that the expression of the TPS gene under low-temperature stress changed significantly, and the expression characteristics of rice and wheat under low-temperature stress were different (Figure 8). Interestingly, the expression levels of some wheat TPS genes (e.g., *TraesCS6B02G091500*, *TraesCS6B02G048600*, and *TraesCS6B02G032300*) were upregulated by low-temperature stress, suggesting these TPS genes’ potential value in wheat cold-tolerance breeding, but the specific biological functions still require further exploration.

## 4. Materials and Methods

### 4.1. Data Sources and Identification of TPSs in Triticum Plants

The reference genome data of the nine species involved in this study were downloaded from the Ensembl Plants and Phytozome databases. The AtTPSs and OsTPSs protein sequences were downloaded from TAIR and the Rice Genome Annotation Project, respectively. The hidden Markov Model (HMM) files of the metal-binding domain (PF01397), C-terminal metal-binding domain (PF03936), and N-terminal domain (PF19086) were downloaded from the InterPro database [30]. HMMER 3.0 (E-value <= 1 × 10^−5^, similarity > 50%) was used to search for TPS proteins from *Triticum* plants [31]. Furthermore, based on the BLASTP method [32], we searched the *Triticum* plants’ protein sequences using AtTPS and OsTPS protein sequences (E-value <= 1 × 10^−5^, similarity > 50%). All candidate TPS protein sequences were used to verify the TPS domain, as analyzed previously [12]. The longest transcript was filtered using the R package seqfinder (https://github.com/yueliu1115/seqfinder, accessed on 24 July 2024).

### 4.2. Phylogenetic Analysis of TPS Genes in Triticum Plants

The Muscle 3.8.1551 software [33] was used to perform multiple sequence alignments of the identified TPS proteins. Then, the maximum likelihood phylogenetic tree was constructed using the IQ-TREE 2.0.3 software [16]. The R package ggtree was used to visualize the phylogenetic tree [17].

### 4.3. Analysis of Cis-Elements in the Promoter of TPS Genes

The 2 kb promoter region sequences upstream of the TPS gene were extracted using a Python program and submitted to the PlantCare database for cis-element prediction [18]. All results were visualized in R 4.3.0 software.

### 4.4. Analysis of Chromosome Distribution, Gene Duplication Events, and Selection Pressure

The chromosomal distribution information of TPS genes of each plant was derived from the genome annotation information of each species. MCScanX 0.8 software was used for the gene duplication and collinearity analysis [34]. The duplicate_gene_classifier program was used to analyze the duplication type of genes. Common gene duplication types include singleton, dispersed duplication (DSD), proximal duplication (PD), tandem duplication (TD), and whole-genome duplication (WGD) [34]. JCVI 1.4.21 software was used for the interspecies collinearity analysis and visualization [35]. ClustalW2.1 software was used to align the protein sequences and CDS sequences of TPS genes with gene duplication [36]. KaKs_Calculator 2.0 software was used to calculate the synonymous substitution rate (synonymous, Ks), nonsynonymous substitution rate (nonsynonymous, Ka), and evolutionary ratio (Ka/Ks) between TPS gene duplicate gene pairs [37].

### 4.5. TPS Protein Interaction Network and Its Functional Annotation

The TPS protein interaction network of the *Triticum* plants was predicted based on the AraNet2 database [19]. The eggNOG-mapper database was used to perform GO functional annotations on the plants involved in this study [20], and R 4.3.0 software was used for the statistics and visualization.

### 4.6. Expression Pattern Analysis of TPSs Using RNA-Seq

The transcriptome data of rice and wheat under the low-temperature treatment were downloaded from the NCBI-SRA database: PRJNA772921 [38] and PRJNA787922 [39]. The transcriptome data were aligned and quantified using the genome alignment software Hisat2 2.2.0 software [40] and featurecount 1.6.4 software in subread [41]. The expression data were visualized using the R package Pheatmap 1.0.12 [42].

## 5. Conclusions

In this study, 531 TPS gene family members in *Triticum* plants were identified and grouped into five subfamilies. As the genome size of the *Triticum* plants expanded, the number of TPS family members also increased. Then, we found that TD and WGD were the major driving forces for the expansion of the TPS family. It is worth noting that as the number of TPSs gradually increased, the protein interaction networks produced became more complex. In addition, RNA-seq data analysis showed that the TPS genes of rice and wheat were expressed under low-temperature stress and showed significantly different expression patterns. Taken together, our results provide a research foundation for further exploration of the TPS genes’ functions in *Triticum* plants and even other plants.

## Data Availability

Data are contained within this article or the Supplementary Materials.

## Supplementary Materials

The following supporting information can be downloaded from https://www.mdpi.com/article/10.3390/ijms25158546/s1.
