# Supplementary figures and images for: Genome-Wide Identification and Evolution-Profiling Analysis of TPS Gene Family in Triticum Plants
Source: Int J Mol Sci. 2024 Aug 5;25(15):8546. doi: 10.3390/ijms25158546 (PMC11312503; doi:10.3390/ijms25158546)

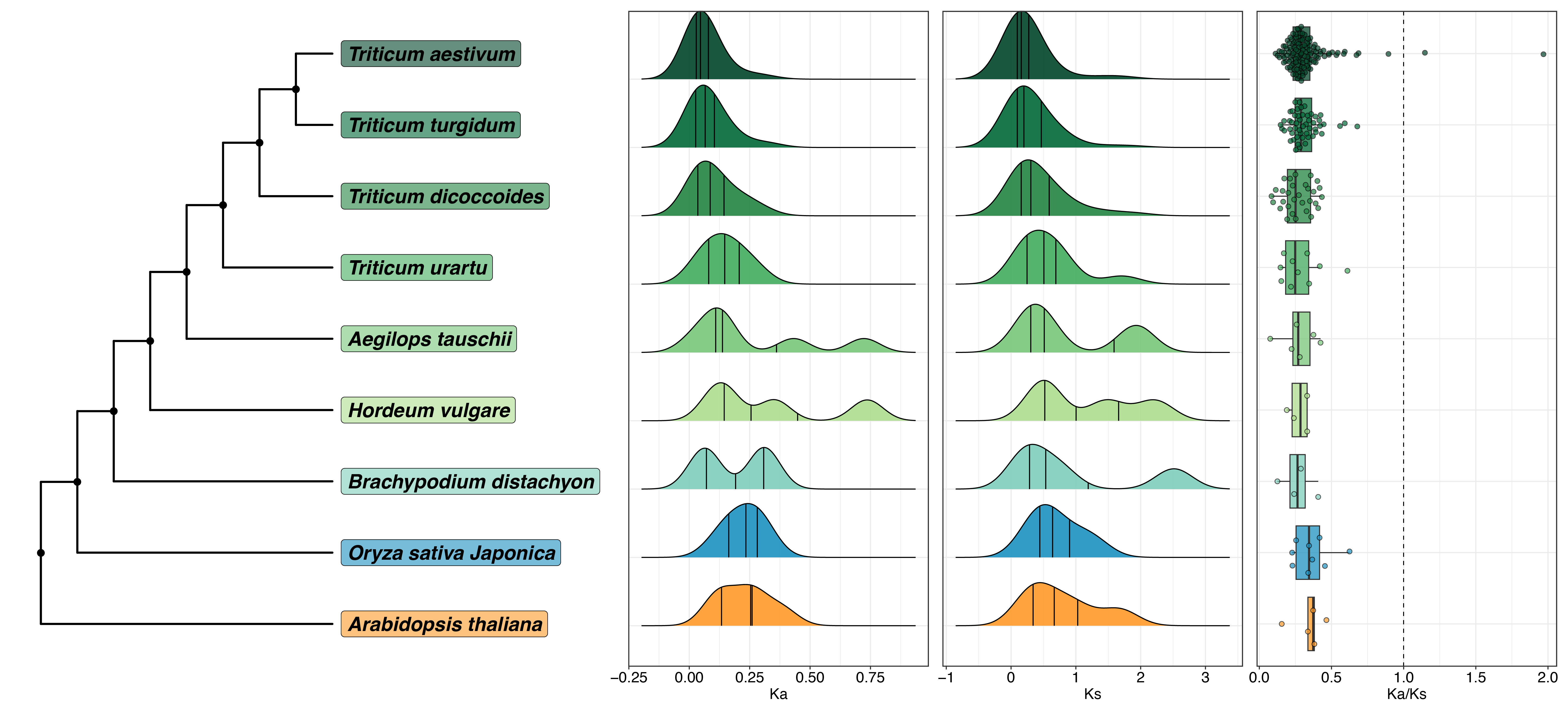

Supplement: Supplementary file 1 [file ijms-25-08546-s001.zip › ijms-3109615-supplementary/supplementary file/Supplemental Figure S1.png]

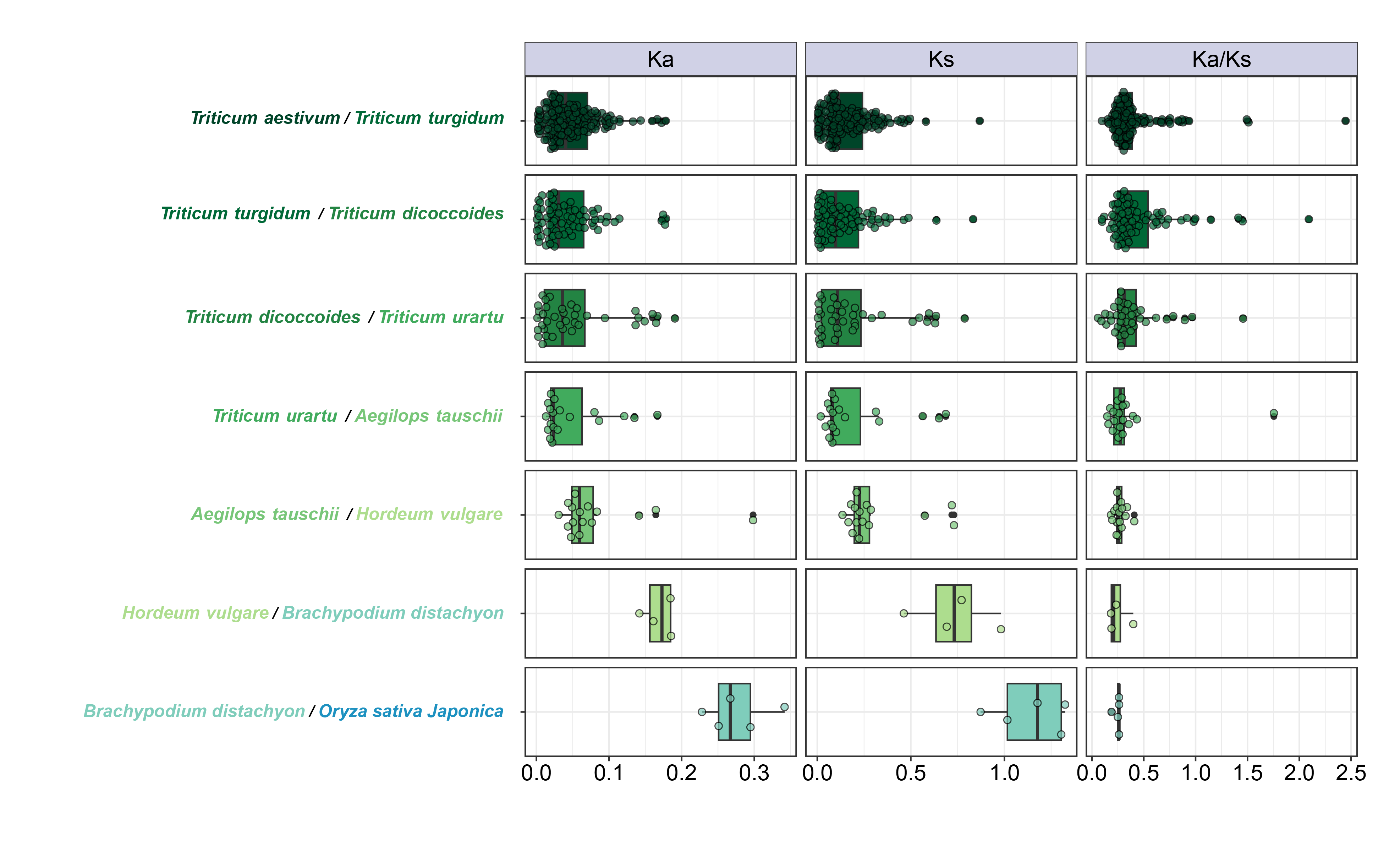

Supplement: Supplementary file 1 [file ijms-25-08546-s001.zip › ijms-3109615-supplementary/supplementary file/Supplemental Figure S2.png]
